# Polymerase ζ Is Involved in Mitochondrial DNA Maintenance Processes in Concert with APE1 Activity

**DOI:** 10.3390/genes13050879

**Published:** 2022-05-13

**Authors:** Heike Katrin Schreier, Rahel Stefanie Wiehe, Miria Ricchetti, Lisa Wiesmüller

**Affiliations:** 1 Department of Obstetrics and Gynecology, Ulm University, 89075 Ulm, Germany; heike.schreier@uni-ulm.de (H.K.S.); rahel.wiehe@gmail.com (R.S.W.); 2 Department of Developmental and Stem Cell Biology, Institute Pasteur, CEDEX 15, 75724 Paris, France; miria.ricchetti@pasteur.fr

**Keywords:** polymerase ζ, mitochondrial DNA degradation, oxidative damage, base excision repair

## Abstract

Mitochondrial DNA (mtDNA) damaged by reactive oxygen species (ROS) triggers so far poorly understood processes of mtDNA maintenance that are coordinated by a complex interplay among DNA repair, DNA degradation, and DNA replication. This study was designed to identify the proteins involved in mtDNA maintenance by applying a special long-range PCR, reflecting mtDNA integrity in the minor arc. A siRNA screening of literature-based candidates was performed under conditions of enforced oxidative phosphorylation revealing the functional group of polymerases and therein polymerase ζ (POLZ) as top hits. Thus, POLZ knockdown caused mtDNA accumulation, which required the activity of the base excision repair (BER) nuclease APE1, and was followed by compensatory mtDNA replication determined by the single-cell mitochondrial in situ hybridization protocol (mTRIP). Quenching reactive oxygen species (ROS) in mitochondria unveiled an additional, ROS-independent involvement of POLZ in the formation of a typical deletion in the minor arc region. Together with data demonstrating the localization of POLZ in mitochondria, we suggest that POLZ plays a significant role in mtDNA turnover, particularly under conditions of oxidative stress.

## 1. Introduction

Mitochondria are important for energy (ATP) production in human cells. These organelles harbor their own double-stranded genome, which encodes 13 proteins involved in oxidative phosphorylation (OXPHOS), 22 tRNAs, and 2 rRNAs. Copy numbers of mitochondrial DNA (mtDNA) differ between cell types, and mtDNA can be in a homoplasmic or heteroplasmic state. If a pathogenic, heteroplasmic mutation reaches a certain threshold, deficiencies in the respiratory-chain emerge [1,2,3]. The origin of heavy strand replication (O_H_) and the origin of light strand replication (O_L_) divide the mitochondrial genome into the major and minor arc [4]. Mitochondrial DNA replication is conducted by a group of proteins that are all encoded in the nucleus. The replicative polymerase in mitochondria is DNA polymerase γ (POLG). The POLG holoenzyme consists of a catalytic subunit (POLGA) and two accessory subunits (POLGB). The catalytic POLGA subunit also harbors a 3′-5′ exonuclease activity [4,5,6]. The mitochondrial replisome consists of POLG and the mitochondrial helicase TWINKLE, which unwinds mtDNA in 5´to 3´direction [7], mitochondrial RNA polymerase (POLRMT), which synthesizes primers for mtDNA replication [8], and mitochondrial single-stranded DNA binding protein (mtSSB), which stimulates POLG processivity [9], TWINKLE helicase activity [10], and is required for replication initiation [11].

Errors in mtDNA replication can result in the loss of whole parts of the mitochondrial genome. If mtDNA replication stalls at direct repeat regions, mis-annealing of these regions can lead to replication slippage, degradation, and deletion of the formed loop [2,12]. The so-called copy-choice recombination provides an alternative mechanism for deletion formation [13]. These processes have been investigated best for the major arc region, where the single-strandedness of the H-strand in the strand-displacement model of replication promotes secondary structure formation during replication [2,4,13].

Except for erroneous replication, deletions can be formed during the repair of damaged mtDNA. The primary repair pathway in mitochondria is base excision repair (BER). Due to the close proximity of the mtDNA to the electron transport chain (ETC), the mtDNA is more susceptible to oxidative DNA damage than nuclear DNA. To remove the damaged base, mitochondrial BER relies on the same steps as nuclear BER: recognition and removal of the damaged base (glycosylases), incision at the abasic site (APE1), gap filling (POLG or POLB), and ligation (LigIII) [14,15,16]. Impaired BER activity as well as the accumulation of oxidative DNA damage and deletions have been linked to aging processes [17,18]. 

In addition to oxidative lesions, double-stranded DNA breaks (DSB) have been reported in the mitochondrial genome [19]. Some proteins of nuclear DSB repair pathways have also been localized in mitochondria (e.g., RAD51, MRE11, DNA2), but it is still unclear whether DSB repair takes place in mitochondria [2]. Due to the multicopy nature of the mitochondrial genome, mtDNA molecules harboring DSBs are degraded rather than repaired [19]. Proteins of the replication machinery (e.g., POLG and MGME1) are involved in the rapid degradation of linear mtDNA [20,21]. If DSBs in mtDNA persist, rearrangements can occur, leading to mtDNA deletions [2,21,22,23]. Increased oxidative stress (ROS) leads to an increase in mtDNA degradation, which is a useful mechanism for preventing the accumulation of damaged copies [24,25]. To cope with the loss of DNA by degradation, the replication of intact mtDNA molecules is compensatorily enhanced [2].

Endonuclease G (ENDOG), a nuclear encoded enzyme that localizes to mitochondria, is involved in nuclear DNA cleavage during early apoptotic processes [26], limited breakage during stress [27], and cleavage of the leukemia-associated breakpoint cluster region in the *Mixed Lineage Leukemia* gene (*MLL*), which requires activity of the BER protein APE1 [28]. Our group previously showed that ENDOG also cleaves mtDNA in the minor arc region. This cleavage was found to depend on ROS, whereby ENDOG-mediated mtDNA depletion led to upregulation of compensatory mtDNA replication [29]. Although an increasing number of proteins with a potential involvement in mtDNA regulation has been reported [30,31,32], the mechanisms of mtDNA repair, DNA degradation, and DNA maintenance are not fully understood. 

To obtain a more comprehensive picture of the different players in mtDNA maintenance, we siRNA screened selected genes for their influence on the stability of the so far underexplored region of mtDNA encompassing the minor arc. Unexpectedly, we identified a destabilizing role of all DNA polymerases with proposed functions in mitochondria, aside from mitochondrial nucleases, including ENDOG. Polymerase ζ (POLZ), the top hit in this group, is a well-known translesion synthesis (TLS) polymerase that can bypass lesions in the nucleus where replicative polymerases will stall [33]. More recently, it has also been shown to localize to mitochondria and influence mtDNA mutability in yeast [34,35] and in human cells [36]. Our work describes new roles of POLZ in balancing repair and degradation of oxidatively damaged mtDNA, and in deletion formation dependent on oxidative stress in mitochondria. 

## 2. Materials and Methods

### 2.1. Cell Culture

Human HeLa cells (provided by Heinrich-Pette-Institute, Hamburg, Germany) were cultivated in high glucose Dulbecco’s Modified Eagle Medium (Gibco/Thermo Fisher Scientific, Waltham, MA, USA) containing 4 mM L-glutamine and 25 mM glucose and supplemented with 10% fetal bovine serum (FBS) (Biochrom, Berlin, Germany). For experiments, HeLa cells were seeded and cultured in DMEM containing no glucose, no glutamine, and no phenol red (Gibco/Thermo Fisher Scientific, Waltham, MA, USA), but supplemented with 10% FBS (Biochrom, Berlin, Germany), 4 mM L-glutamine (Gibco/Thermo Fisher Scientific, Waltham, MA, USA), and 50 mM galactose (Genaxxon bioscience GmbH, Ulm, Germany) for 72 h. The indicated cells were cultured in high glucose DMEM with FBS even for experiments. All cells were grown at 37 °C with 5% CO_2_. 

### 2.2. siRNA Transfection

For siRNA-mediated knockdown of different proteins in HeLa cells HiPerfect^®^ (Qiagen, Hilden, Germany) was used. Therefore, cells were transfected with 1 nM FlexiTube Gene-Solution (Qiagen, Hilden, Germany), which provides a mix of four distinct siRNAs for the indicated proteins, as successfully pre-established in Gole et al., 2018 and Wiehe et al., 2018 (Supplementary Table S1). Non-silencing RNA (nsRNA) (Qiagen, Hilden, Germany) was engaged in negative controls. 24 h after transfection, the medium was replaced and the cells were cultivated for a further 24 h. 

### 2.3. Treatments

HeLa cells were treated with 15 µM APE1 inhibitor III (APE1inhIII) (Merck Millipore, Burlingtion, MA, USA) or the solvent DMSO (Merck Millipore, Burlingtion, MA, USA) for 5 h at 37 °C [28] before mitochondrial replication imaging protocol (mREP) or total DNA isolation was performed. For scavenging of ROS, HeLa cells were treated for 24 h with either 100 µM mitoTempo (Sigma-Aldrich/Merck, St. Louis, MO, USA) [37] or N-acetyl-L-cysteine (NAC) (Sigma-Aldrich/Merck, St. Louis, MO, USA) [29,38,39,40] or water as solvent. To block mitochondrial import of proteins, HeLa cells were treated for 48 h with 10 µM mitoBlock-6 (Focus Biomolecules, Plymouth Meeting, PA, USA). To slow down mitochondrial DNA replication, the HeLa cells were treated for 24 h with 10 µM 2′,3′-Dideoxycytidine (ddC) (Sigma-Aldrich/Merck, St. Louis, MO, USA) [41].

### 2.4. Total Genomic DNA Isolation

Total genomic DNA isolation (including mitochondrial DNA) was performed with the QIAamp^®^ DNA Mini Kit (Qiagen, Hilden, Germany) following the manufacturer’s instructions. The amount of total DNA was quantified using a NanoDrop 2000 Spectrometer (Thermo Fisher Scientific, Waltham, MA, USA).

### 2.5. Long-Range PCR

To amplify the complete mitochondrial genome, two sets of primers (Set1 and Set2) were used to perform a special long-range PCR [42]. Set1 (forward primer, 5′-GCAACCTTCTAGGTAACGACC-3′; reverse primer, 5′-GAGTCAATACTTGGGTGGTAC-3′) and Set2 (forward primer, 5′-CATTGGACAAGTAGCATCCGT-3′; reverse primer, 5′-GCCTCCGATTATGATGGGTAT-3′) regions were amplified from 20 ng of total DNA for initial denaturation at 94 °C for 1 min followed by 23–28 cycles of 94 °C 30 s, 56 °C 45 s, 68 °C 11 min and for final elongation 72 °C for 10 min with LA Taq^®^ DNA Polymerase (TaKaRa Bio Inc, Kusatsu, Japan). A shorter fragment in the 12S region of mtDNA (forward primer, 5′-CCCCCTCCCCAATAAAGCTAAAACT-3′; reverse primer, 5′-TTTTAAGCTGTGGCTCGTAGTGT-3′) was amplified with my-Budget Taq-DNA-Polymerase (Bio-Budget Technologies GmbH, Krefeld, Germany) and run at 95 °C for 5 min for initial denaturation followed by 21 cycles of 95 °C 90 s, 60 °C 60 s, 72 °C 30 s and 72 °C for 7 min for final elongation and used for normalization to total mtDNA. PCR products were loaded on 0.5% (Set1 and Set2) or 2.5% (12S) agarose gels and bands were imaged by ChemiDoc™ MP (Bio-Rad Laboratories, Hercules, CA, USA). The band intensity was quantified in the linear range of detection using Image Lab™ (Bio-Rad Laboratories, Hercules, CA, USA) software.

### 2.6. Del3895 PCR

For detection of the 3895 bp deletion, the PCR product Del3895 (forward primer, 5′-CTTTTGGCGGTATGCACTTT-3′; reverse primer, 5′-GATTATGGATGCGGTTGCTT-3′) was amplified from 100 ng of total DNA with my-Budget Taq-DNA-Polymerase (Bio-Budget Technologies GmbH, Krefeld, Germany) with initial denaturation at 94 °C for 10 min followed by 39 cycles of 94 °C 30 s, 56 °C 30 s, 72 °C 30 s and final elongation at 72 °C for 7 min. The short elongation time of 30 s will not allow the amplification of the wild-type PCR product; instead, just the short 375 bp product generated by deletion of the 3895 bp fragment will be amplified [43]. Amplified products were separated by 2.5% agarose gel, imaged by ChemiDoc™ MP (Bio-Rad Laboratories, Hercules, CA, USA), and quantified by Image Lab™ (Bio-Rad Laboratories, Hercules, CA, USA) software.

### 2.7. Immunofluorescence Staining

HeLa cells were cultured on glass slides, fixed with 2% paraformaldehyde (Electron Microscopy Sciences, Hatfield, PA, USA) for 20 min, permeabilized with 0.5% Triton X-100 (Sigma-Aldrich/Merck, St. Louis, MO, USA), and unspecific binding was prevented by blocking with 5% goat serum. The slides were incubated overnight at 4 °C with the primary antibodies (rabbit-anti-human REV3L (Lifespan Biosciences, Seattle, WA, USA, LS-C368491) and mouse-anti-human Tom22 (Santa Cruz Biotechnology, Dallas, TX, USA, sc-58308). As secondary antibodies, Alexa Fluor 488 anti-rabbit, Alexa Fluor 555 anti-mouse, and Alexa Fluor 555 anti-rabbit (all from Invitrogen/Thermo Fisher Scientific, Waltham, MA, USA) were used for 45 min at 37 °C. Nuclei were stained with 1 mM DAPI for 7 min, and slides were mounted with Mowiol-Dabco. Images were taken either by a Keyence BZ-9000 microscope (Keyence, Neu-Isenburg, Germany) or LSM710 (Carl Zeiss AG, Oberkochen, Germany). The fluorescence intensity was determined with ImageJ software (ImageJ, U.S. National Institutes of Health, Bethesda, MD, USA).

### 2.8. MitoTracker Staining 

Mitochondria were stained by incubation at 37 °C for 45 min with 250 nM MitoTracker^®^ Deep Red (DR) FM (Life technologies™/Thermo Fisher Scientific, Waltham, MA, USA) and further used for immunofluorescence staining.

### 2.9. mTRIP Imaging Protocol

mTRIP with the replication probes (mREP) was performed as previously described [44,45,46]. mREP (marker for mitochondrial DNA replication initiation) probes were amplified by PCR (forward primer, 5′-ACATTATTTTCCCCTCCC-3′; reverse primer, 5′-GGGGTATGGGGTTAGCAG-3′). The mREP probes were labeled with fluorescence using the NT labeling kit Atto555 or Atto647 (Jena Bioscience, Jena, Germany). Cells were cultured on glass slides, hybridized with mREP probe, washed, stained with 1 mM DAPI for 7 min, mounted with Mowiol–Dabco, and imaged using LSM710 (Carl Zeiss AG, Oberkochen, Germany). Relative fluorescence intensities were determined with ImageJ software (ImageJ, U.S. National Institutes of Health, Bethesda, MD, USA).

### 2.10. Proximity Ligation Assay (PLA)

HeLa cells were cultured on glass slides and fixed with 2% PFA for 20 min. The staining of the PLA was performed following the manufacturer’s instructions with Duolink™ In Situ Detection Reagent Orange, Duolink™ In Situ PLA Probe Anti-Rabbit PLUS, Duolink™ In Situ PLA Probe Anti-Mouse MINUS, and Duolink™ In Situ Mounting Medium with DAPI (Sigma-Aldrich/Merck, St. Louis, MO, USA). As primary antibodies, rabbit-anti-human REV3L (Lifespan Biosciences, Seattle, WA, USA, LS-C368491), mouse-anti-human EndoG (Santa Cruz Biotechnology, Dallas, TX, USA, sc-365359), mouse-anti-human DNA POLγ (Santa Cruz Biotechnology, Dallas, TX, USA, sc-3906349), rabbit-anti-human DNA POLγ (Cell Signaling Techonolgy Inc., Danvers, MA, USA, 13609S) mouse-anti-human TLR4 (Santa Cruz Biotechnology, Dallas, TX, USA, sc-293072), and mouse-anti-human mtTFA (Santa Cruz Biotechnology, Dallas, TX, USA, sc-166965) were used. Images were taken with a Keyence BZ-9000 microscope (Keyence, Neu-Isenburg, Germany) and foci were counted manually. 

### 2.11. Quantitative Reverse Transcription Polymerase Chain Reaction (qRT-PCR)

Total RNA was isolated from HeLa cells according to the manufacturer’s instructions with the RNeasy Plus Mini Kit (Qiagen, Hilden, Germany). A QuantiTec Reverse Transcription Kit (Qiagen, Hilden, Germany) was used for reverse transcription of 1 µg of total RNA. qPCR was performed with the qTower (Analytik Jena, Jena, Germany) using the program 2 min 95 °C and 40 cycles of 10 s 95 °C and 20 s of 60 °C. The genes of interest were analyzed with EndoG-FAM, PolGA-FAM, and REV3L-FAM (Bio-Rad Laboratories, Hercules, CA, USA). As controls, PPIA-HEX, GAPDH-HEX (Bio-Rad Laboratories, Hercules, CA, USA), and B2M-VIC (Thermo Fisher Scientific, Waltham, MA, USA) were utilized. 

### 2.12. Statistical Analysis

All graphs were created using GraphPad Prism 9 (GraphPad Software, San Diego, CA, USA). Outliers were identified and removed by the ROUT method (Q = 0.1%). All experiments were conducted independently at least twice. The *p* values of unpaired, nonparametric samples were determined using the Mann–Whitney U test (* *p* ≤ 0.05, ** *p* ≤ 0.01, *** *p* ≤ 0.001, **** *p* ≤ 0.0001).

## 3. Results

### 3.1. Identification of Proteins Involved in mtDNA Maintenaince

**Figure 1:**
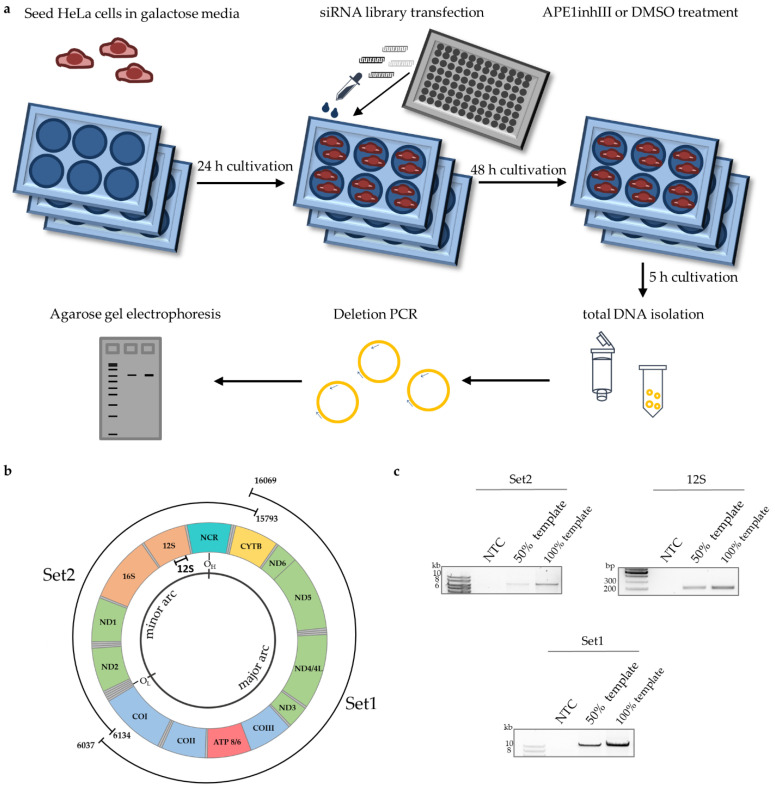
Screening for proteins involved in mtDNA maintenance. Schematic representation of experimental design for siRNA screening of a library comprising 57 siRNAs and nsRNA control (**a**), scheme of mitochondrial long-range PCR for amplification of whole mitochondrial genome; NCR is for the non-coding (regulatory) region (**b**) and representative agarose gels of amplified mitochondrial regions (Set1, Set2, 12S) confirming analysis in the linear range of PCR (no template control (NTC), 50% template, and 100% template) (**c**).

Previously, we discovered the involvement of ENDOG in the regulation of mtDNA maintenance through nucleolytic attack within the so-called Set2 region of the mitochondrial genome, encompassing terminally replicated mtDNA between the origins of light strand and heavy strand replication, O_L_ and O_H_ [29]. Notably, the Set1 region covering the mtDNA that was replicated before Set2 was not cleaved by ENDOG [29]. To identify new players in this process, siRNA screening was performed (Figure 1a). Therefore, we assembled an siRNA library targeting 57 candidates (Supplementary Table S1). These candidates were selected because they were known or predicted to localize to mitochondria and/or to impact mitochondrial genome stability. The investigated proteins are (potentially) involved in different DNA repair pathways of relevance for mitochondria [47,48], such as base excision repair (BER), DSB repair or single-strand break (SSB) repair, in mtDNA replication, in RNA regulatory processes in mitochondria (e.g., in mtDNA transcription), or in executing enzymatic functions related to these processes. 

For screening, HeLa cells were seeded in a 6-well plate in galactose instead of glucose medium, as galactose medium more closely mimics the physiological situation than cultivation in high-glucose medium. Galactose channels energy production from glycolysis to OXPHOS, leading to elevated endogenous ROS levels in the cells [49,50]. This aspect was critical to unveiling the role of ENDOG in mtDNA cleavage in our previous study [29]. For knockdown, cells were transfected with gene-specific siRNA pools (4 siRNAs each), cultured for 48 h, and afterwards treated for 5 h with DMSO or APE1 inhibitor III (APE1inhIII). APE1 is central to the main repair pathway in mitochondria, namely BER [16]. Finally, total DNA was isolated, and a pre-established long-range PCR was performed (Figure 1b) [42]. To ensure that the mtDNA was amplified in the linear range of the PCR, we performed a 50% template control along with a 100% template control in each experiment (Figure 1c). For screening factors involved in mtDNA maintenance, we amplified the Set2 region of the mitochondrial genome (long fragment) and normalized it to a short fragment within the 12S gene, for assessment of the amount of fully synthesized mtDNA.

**Figure 2:**
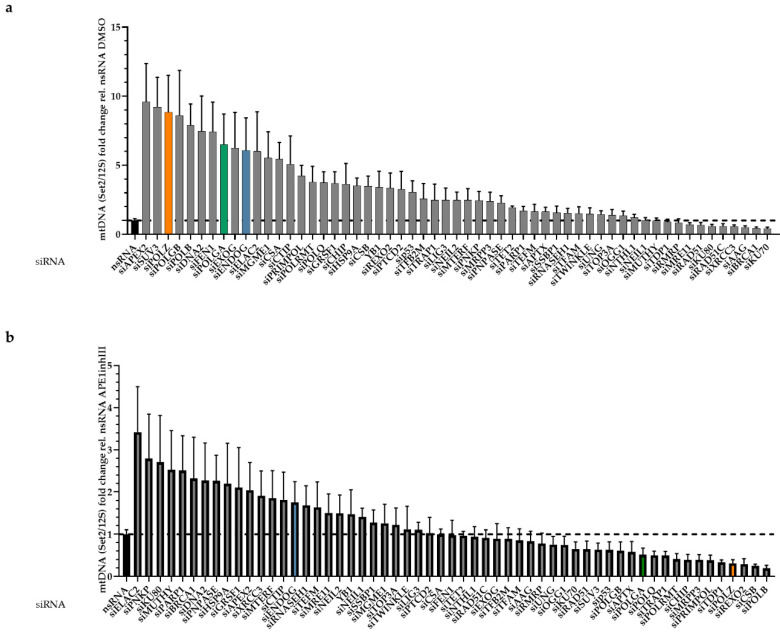
Waterfall plot of mtDNA changes in the siRNA screen. HeLa cells were grown in galactose medium and subjected to siRNA-mediated knockdown of the indicated candidate proteins associated with mitochondrial functions for 48 h. Cells were treated with DMSO (**a**) or 15 µM APE1inhIII (**b**) for 5 h. PCR products of the 7 kb long Set2 mtDNA region amplified from total DNA were quantified, normalized to the sample-specific values for the 0.2 kb short mitochondrial 12S region and plotted relative (rel.) to the mean values for nsRNA transfected samples per experiment (*n* = 6–8 from 4 independent experiments). All values are means ± SEM. Note that colored bars indicate data obtained after knockdown of high priority factors of this study further on, namely blue bars after ENDOG knockdown, green bar after POLGA knockdown, and orange bar after POLZ knockdown.

Following PCR amplification, mtDNA band intensities were densitometrically quantified, and the results obtained after 4 screening rounds, i.e., results from 4 independent experiments, were depicted in a waterfall plot. For reasons of clarity, protein names were used throughout this manuscript although siRNAs target mRNAs of genes. The respective gene names are listed in Supplementary Table S1. The knockdown of most of the factors led to increased levels of Set2 mtDNA (Figure 2a). In the presence of APE1inhIII, i.e., under conditions of BER inhibition, simultaneous knockdown of the majority of proteins led to a decrease in the Set2 mtDNA level (Figure 2b). 

**Figure 3:**
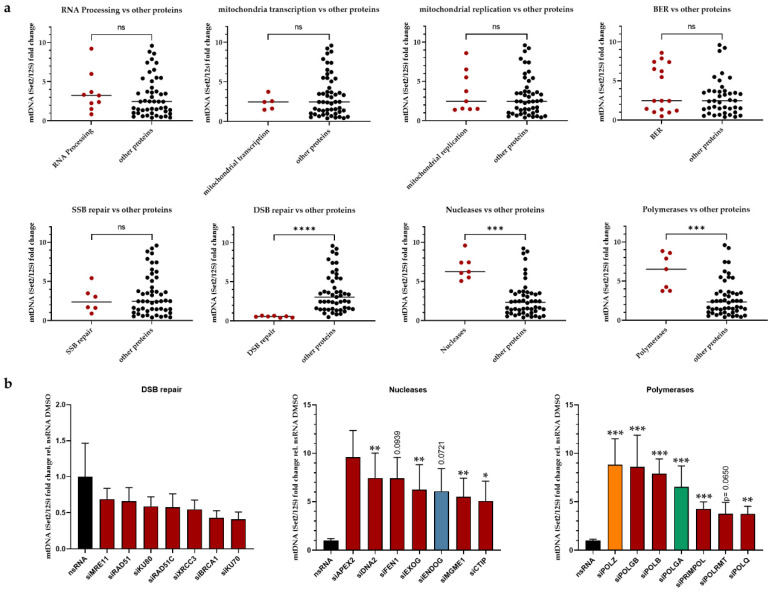
Analysis of functionally different groups. Mean mtDNA (Set2/12S fold change rel. to nsRNA) values for candidates included in the siRNA screening were functionally grouped and compared to the group of remaining candidates to identify the most significantly altered function (**a**). For individual candidates from significantly altered groups, individual differences compared to the control (nsRNA) were calculated (*n* = 6–8 from 4 independent experiments) (**b**). For group annotations, see Supplementary Table S1. All values are means ± SEM (* *p* ≤ 0.05, ** *p* ≤ 0.01, *** *p* ≤ 0.001 and **** *p* ≤ 0.0001); ns, not significant.

To obtain better insight into the results of the siRNA screening, the investigated proteins were grouped according to the pathway in which they are involved and/or their biochemical functions, and compared to all other investigated proteins. Of note, the average values obtained for the group of DSB repair proteins were remarkably lower than the other proteins, whereas they were higher for the groups of polymerases and nucleases (Figure 3a). For all other functional groups, no significant differences were observed. To further delineate the impact of individual proteins in the three groups with identified differences in Set2 mtDNA content, the mean values obtained after knockdown of single members of these groups were compared to the respective nsRNA control. Knockdown of none of the DSB repair proteins caused a significantly different Set2 mtDNA level (Figure 3b, left panel). Conversely, and as expected for the group of nucleases, which are known to attack the integrity of mtDNA, Set2 levels significantly rose or showed this trend (*p* < 0.1) upon knockdown of 6 out of 7 tested candidates, namely DNA2, FEN1, EXOG, ENDOG, MGME1, and CTIP (Figure 3b, middle panel). Unexpectedly, a significant increase in Set2 mtDNA levels was also seen upon knockdown of 6 out of 7 polymerases or polymerase subunits. The only exception was the mitochondrial RNA polymerase (POLRMT) (*p* = 0.0650), suggesting that the loss of DNA polymerases protects Set2 mtDNA (Figure 3b, right panel). Both subunits of polymerase γ (POLG), which is the replicative polymerase in mitochondria, were hits of this group. Of interest, POLGA, the catalytic subunit of POLG, also increases the lifetime of error-containing mtDNA [21]. The activity of the 3′-5′ exonuclease [51] is necessary for the degradation of mtDNA fragments [21]. Downregulation of POLGA may therefore increase the lifetime of error-containing mtDNA [21].

However, in the polymerase group, the top hit was POLZ, which is devoid of a proofreading activity [52]. It is still under debate whether POLZ localizes to mitochondria and plays a functional role in this organelle [53]. Notably, however, there is evidence of a functional mitochondrial localization signal (MLS) enabling POLZ localization to mitochondria [36]. Knockdown of POLZ led to a highly significant 8.8-fold increase in mtDNA band intensity of the Set2 region, suggesting a destabilizing role of POLZ for this region of mtDNA (Figure 3b, right panel). APE1inhIII-treated cells showed a 4.6-fold increase in Set2 mtDNA (*p* ≤ 0.0001) compared to the control cells (Supplementary Figure S1a). Under APE1inhIII treatment, there is no further increase of Set2 mtDNA in the group of nucleases as well as polymerases, suggesting a dependence of mtDNA accumulation on APE1 activity (Supplementary Figure S1b).

### 3.2. Roles of POLZ in mtDNA Stability as a Function of Mitochondrial ROS

**Figure 4:**
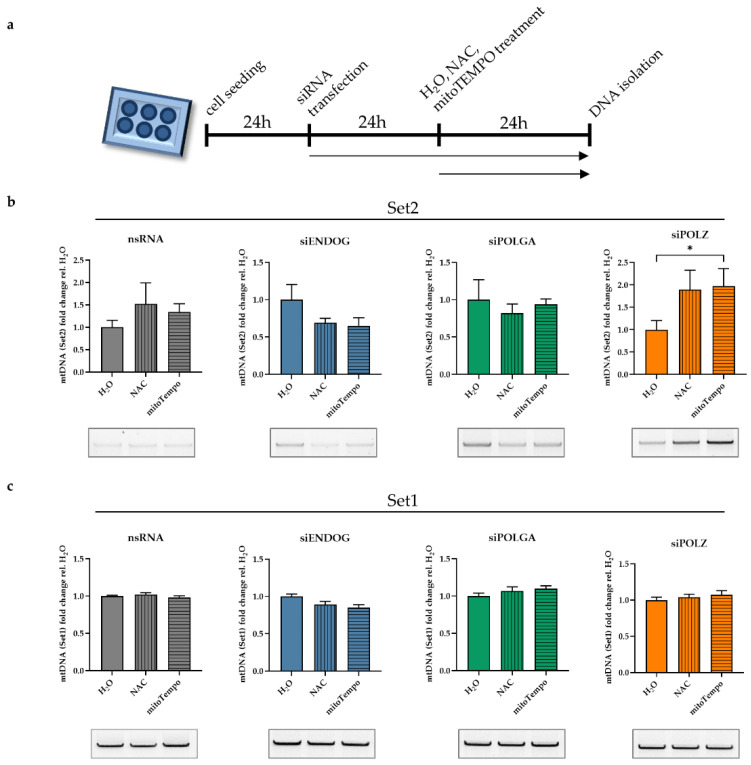
The mitochondrial ROS quencher protects the mitochondrial genome in the absence of POLZ. Hela cells were cultured in galactose medium, and knockdown was performed for 48 h. Total DNA was isolated after treatment with H_2_O, or 2 mM NAC, or 100 µM mitoTempo for 24 h (**a**). Quantified Set2 PCR products were plotted relative to the H_2_O control for cells transfected with nsRNA (gray bars), siENDOG (blue bars), siPOLGA (green bars), or siPOLZ (orange bars) (**b**). Quantified Set1 PCR products were plotted relative to the H_2_O control for cells transfected with nsRNA (gray bars), siENDOG (blue bars), siPOLGA (green bars), or siPOLZ (orange bars) (**c**). Representative agarose gels are shown below the graphs. All values are means ± SEM (*n* = 8–10 of 5 independent experiments) (* *p* ≤ 0.05).

We further investigated the impact of ROS, which is the main DNA damaging source of mtDNA. The resulting oxidative stress is particularly relevant in cells cultivated in galactose medium, such as during siRNA screening [49,50], due to the close proximity of the electron transport chain to the mtDNA [54]. Therefore, we treated cells with either n-acetyl cysteine (NAC), a membrane-permeable antioxidant [55], or mitoTempo, an antioxidant that is directly targeted to mitochondria and scavenges mitochondrial ROS [56]. In the nsRNA transfected control cells, we observed a slight but not significant increase of Set2 mtDNA band intensity upon antioxidant treatment (Figure 4b, gray bars). 

There was no significant difference in Set2 mtDNA detectable after NAC or mitoTempo treatments in either ENDOG (Figure 4b, blue bars) or POLGA knockout cells (Figure 4b, green bars). Conversely, upon POLZ knockdown, NAC and mitoTempo treatments induced elevated Set2 mtDNA levels (Figure 4b, orange bars), although values were significant only after mitoTempo treatment, possibly due to the more efficient ROS scavenging effect of mitoTempo in mitochondria compared to global anti-ROS activity of NAC [57,58].

These effects were detectable in the Set2 region of the mitochondrial genome only, whereas in the Set1 region, no changes in mtDNA levels were observed regardless of the knockdown or the treatment (Figure 4c). The knockdown efficiencies of all the nucleases and polymerases investigated here were investigated by qRT-PCR (Supplementary Figure S2). These results suggest a specific influence of POLZ on the Set2 region content that was amplified after ROS quenching.

### 3.3. POLZ Is Involved in 3895 bp Deletion Formation

**Figure 5:**
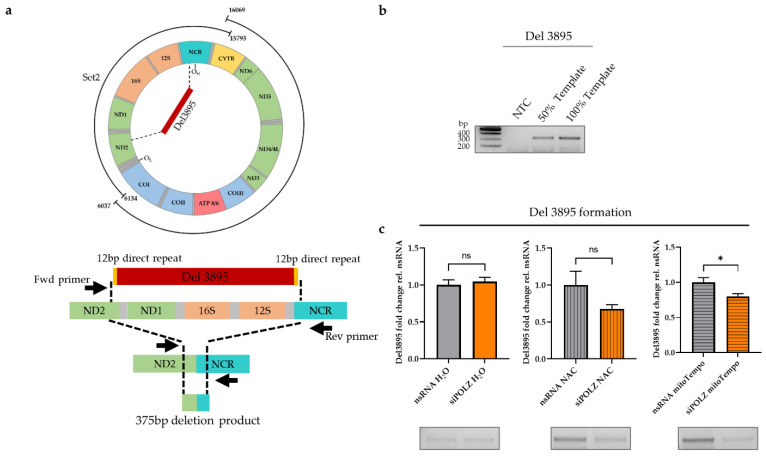
POLZ is involved in the Del3895 formation. Schematic presentation of the location of 3895 bp deletion (**upper**) and the location of primers for amplification of 375 bp generated by the 3895 bp deletion (**lower**) (**a**). Representative agarose gels of 375 bp deletion product confirming analysis in the linear range of PCR (50% template and 100% template) (**b**) and quantification of Del3895 deletion product from HeLa cells cultured in galactose medium, siRNA-mediated knockdown for 48 h plus 24 h treatment with 100 µM mitoTempo or 2 mM NAC (*n* = 10 from 5 independent experiments) (**c**). Representative agarose gels are shown below the graphs. All values are means ± SEM (* *p* ≤ 0.05); ns, not significant.

Since the impact of POLZ on mtDNA stability was observed only in the Set2 region, we investigated the possibility of the formation of deletions within this region. One major deletion in the Set2 region, namely a 3895 bp, has been reported to be UV-induced [43,59,60]. POLZ has been shown to contribute to bypassing UV-induced DNA lesions as a TLS polymerase in the nucleus [61]. The degree of 3895 bp deletion formation can be determined by a special PCR utilizing primers adjacent to the Del3895 region (Figure 5a). Accordingly, we chose a short elongation time of 30 s, limiting amplification to the 375 bp deletion product as compared to a theoretical 4273 bp PCR product encompassing the Del3895 region in the intact mtDNA (Figure 5b). Under conditions of endogenous, i.e., galactose medium-induced oxidative stress, there was no difference in the degree of Del3895 formation between the nsRNA control and POLZ knockdown samples. When ROS was quenched by NAC treatment, we noticed a slight but not significant decrease in Del3895 formation after POLZ knockdown. This effect was more robust in mitoTempo-treated cells, causing a 21% reduction of deletion formation (*p* = 0.0138) (Figure 5c). Importantly, knockdown of ENDOG or POLGA had no effect on Del3895 formation in the presence of antioxidant treatment (Supplementary Figure S3a,b). These results support the notion of a destabilizing effect of POLZ on Set2 mtDNA and suggest that the formation of Del3895 contributes to the POLZ-dependent loss of the integrity of Set2 mtDNA when quenching mitochondrial oxidative stress.

### 3.4. POLZ Knockdown Stimulates mtDNA Replication Initiation

To understand whether these effects of POLZ were linked with perturbations of mtDNA replication, we treated cells with ddC for 24 h. Among the DNA polymerases, POLG is known to discriminate the least against this dideoxynucleotide, which is why ddC has frequently been used to specifically inhibit mtDNA replication [41]. PCR analyses demonstrated that the accumulation of Set2 after POLZ knockdown is lost after ddC treatment, suggesting a requirement of mtDNA replication at normal speed (Supplementary Figure S4a). For comparison, Set1 levels diminished after ddC treatment independently of POLZ knockdown (Supplementary Figure S4b). Del3895 formation was reduced in ddC-treated cells without, but not with, POLZ knockdown (Supplementary Figure S4c). These data suggest that the destabilizing effect of POLZ on Set2 mtDNA and Del3895 formation require an unperturbed replication speed. Of note, previous analysis of nuclear DNA replication has shown that not only blocked but also fast DNA synthesis can cause replication stress [62]. Moreover, the same ddC treatment was reported to reduce OXPHOS (reviewed in [41]). Altogether, these findings support the notion that ROS and/or replication stress are linked to the destabilizing effect of POLZ on mtDNA.

**Figure 6:**
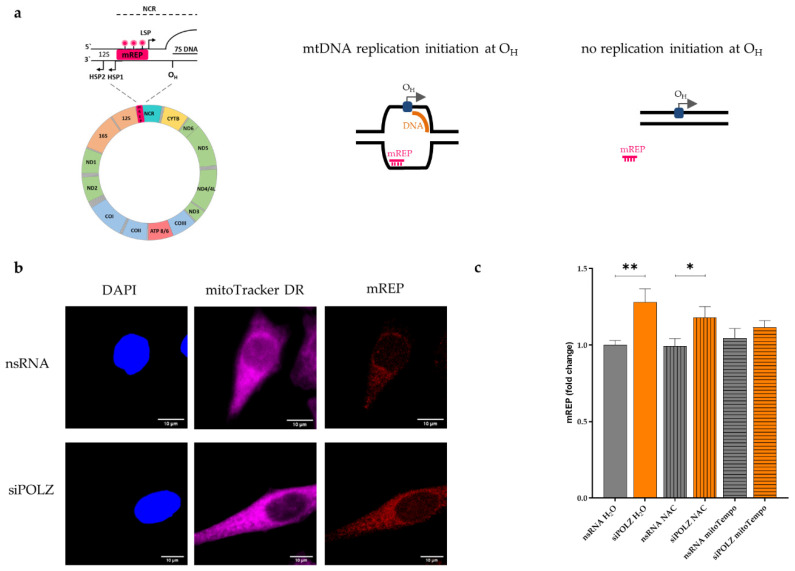
POLZ knockdown leads to compensatory mtDNA replication initiation under conditions of oxidative stress. Scheme of mREP recognition site in mitochondrial genome (**left**, [29]) and mREP recognition of open DNA replication bubble when replication initiation occurs at O_H_ (**right**, [44]) (**a**). Representative confocal microscopic images of the mREP fluorescence signal of HeLa cells cultured in galactose media and siRNA-mediated knockdown for 48 h, scale bar = 10 µM (**b**). DAPI is used as a reference for the nuclear and extra-nuclear surface (mitochondria located over the nuclear surface may also be mREP-positive), and mitoTracker DR was used as a mitochondrial marker. Fluorescence-based quantification of mREP signal, *n* = 35–42 cells from 2 independent experiments, cultured in galactose medium, siRNA-mediated knockdown of POLZ for 48 h versus nsRNA control plus treatment with H_2_O, or 2 mM NAC or 100 µM mitoTempo for 24 h (**c**). All values are means ± SEM (* *p* ≤ 0.05, ** *p* ≤ 0.01).

To examine if POLZ is playing a role in mtDNA replication, we performed an imaging protocol (mTRIP) that specifically measures mtDNA initiation of replication, using the mREP probe [44,45,46]. To quantify initiation of replication, a fluorescence labeled DNA probe was hybridized in situ between the LSP and HSP promotors near the origin of heavy strand replication O_H_ (Figure 6a, left panel). If unidirectional mtDNA replication is initiated at O_H_, the mREP probe can bind to the non-replicated DNA strand, which becomes accessible due to the formation of a replication bubble. This gives rise to a fluorescent signal that can be detected by confocal microscopy (Figure 6b). When there is no replication initiation at O_H_, the mREP probe is unable to bind to the mtDNA, and no fluorescent signal is emitted (Figure 6a, schemes on the right). mREP analysis performed after POLZ knockdown resulted in an increase of the fluorescent signal (1.3-fold, *p* = 0.0026) in the H_2_O treated (control) cells. This effect was also visible in NAC-treated cells (1.2-fold, *p* = 0.0496), but not when mitochondrial ROS was scavenged by mitoTempo treatment (Figure 6c). Compensatory replication of mtDNA triggered by DNA damage has been previously reported [63]. In view of our data obtained in POLZ knockdown cells showing reduced Del3895 formation only in the presence of a mitochondrial ROS scavenger (Figure 5c), and Set2 mtDNA accumulation also without ROS quenchers (Figure 3b) we conclude that POLZ-mediated repression of mtDNA replication initiation associates with Set2 mtDNA accumulation rather than Del3895 formation. Altogether, our data suggest that knockdown of POLZ stimulates compensatory mtDNA replication in the case of mitochondrial oxidative stress. 

### 3.5. POLZ Localizes to Mitochondria 

**Figure 7:**
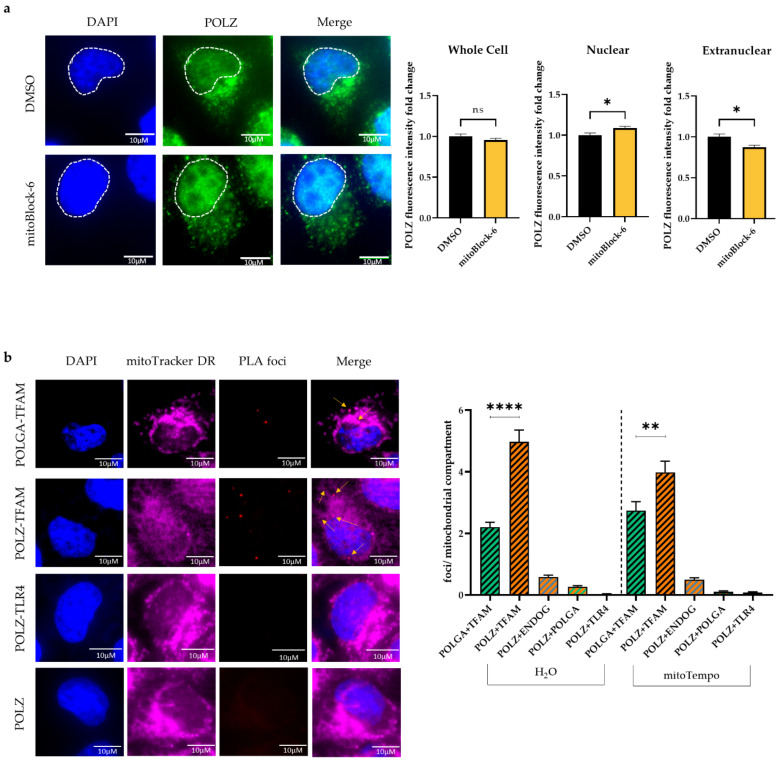
POLZ localizes to mitochondria. Representative images of POLZ fluorescence intensity in HeLa cells treated for 48 h with DMSO or 10 µM mitoBlock-6 (**left**) and their quantification (**right**) of two independent experiments, *n* = 92–99 cells (**a**). Representative images of the proximity ligation assay (PLA, **left**) and the quantification of PLA foci (**right**) of two independent experiments, *n* = 100–183 cells (**b**). All values are means ± SEM (* *p* ≤ 0.05, ** *p* ≤ 0.01, **** *p* ≤ 0.0001), scale bar = 10 µM.

Since there is still a debate on whether POLZ localizes to mitochondria [53] and only a few publications provide evidence for mitochondrial localization of POLZ [34,36], we aimed to further substantiate this aspect. Therefore, we quantified POLZ-specific signals in HeLa cells by immunofluorescence microscopy (Figure 7a). POLZ fluorescent signals were detectable in the nucleus as well as in extra-nuclear regions, showing intense punctate staining suggestive of localization in mitochondria. This notion was strengthened by co-staining with the mitochondrial marker TOM22 (Supplementary Figure S5). Next, we treated cells with a mitochondrial import inhibitor (mitoBlock-6) to reduce the import of proteins into the mitochondria. After mitoBlock-6 treatment, the overall POLZ intensity remained the same, whereas the signal in the nucleus increased in contrast to the extranuclear signal, which decreased (Figure 7a, right panel). These results support the concept that a fraction of the nuclear encoded POLZ protein is transported into mitochondria. As another method to sustain the evidence of mitochondrial POLZ localization, we performed a proximity ligation assay (PLA) to show the close proximity of POLZ to a well-established mitochondrial protein. As a positive control, we examined the localization of POLGA adjacent to TFAM, both well described mitochondrial matrix proteins [6,64], and found formation of distinct PLA foci, as expected (Figure 7b). As a negative control, PLA for POLZ and the membrane receptor TLR4 was performed, and indeed no PLA foci were detected. Interestingly, PLA foci were observed for POLZ and TFAM (Figure 7b). POLZ-TFAM-specific PLA foci numbers were higher than for POLGA and TFAM, strongly supporting mitochondrial localization of POLZ. We also observed a close proximity of POLZ to ENDOG and POLGA, although to a much lesser extent than to TFAM. The formation of POLZ-TFAM-specific PLA foci showed a decrease when the cells were treated with mitoTempo, indicating a more pronounced mitochondrial localization of POLZ under conditions of oxidative stress. However, this trend can be explained by lower POLZ protein levels under mitoTempo treatment (Supplementary Figure S6a,b), which we also observed in cells cultivated in glucose versus galactose medium (Supplementary Figure S6c,d). In Figure 7b punctate POLZ-specific signals outside the nucleus colocalized with mitoTracker DR staining, further supporting mitochondrial localization of this polymerase. We also noticed that POLZ knockdown led to a significant decrease (0.7-fold, *p* ≤ 0.0001) of TOM22 fluorescence intensity, suggesting a reduction in mitochondrial mass after POLZ depletion (Supplementary Figure S7). Taken together, the results obtained using different experimental approaches provide evidence for a localization of a fraction of POLZ in mitochondria and support the notion that POLZ plays a role in mtDNA maintenance under conditions of high as well as low oxidative stress. 

## 4. Discussion

In this study, we searched for proteins involved in mtDNA maintenance regulation, engaging a pre-established long-range PCR for amplification of the so-called Set2 region of mtDNA [36] that essentially corresponds to the minor arc. Our previous work revealed that this part of mtDNA is especially cleaved by ENDOG during conditions of oxidative stress [29]. Here, with an siRNA screening of 57 proteins, previously suggested to execute different mitochondrial DNA- or RNA-related functions, we identified several new candidates to reduce mtDNA integrity. The identified proteins with the most pronounced influences belong to the groups of nucleases and polymerases.

Among the groups of identified nucleases, CTIP is best known for its functions in DSB resection in the nucleus, where it promotes HR and microhomology-mediated end-joining (MMEJ) [65]. In mitochondria, CTIP has been shown to participate in MMEJ [66]. MMEJ can lead to large-scale deletions both in the nucleus and in mitochondria [66,67]. Thus, the increased levels of Set2 after CTIP knockdown could be explained by diminished MMEJ and consequently fewer deleterious mtDNA rearrangements in this region.

The nuclease DNA2 is well known for its nuclear activity in processing DNA regions difficult to replicate (e.g., G-quadruplex) to prevent persistent stalling of replication forks [68]. In mitochondria, DNA2 has been shown to interact with POLG and stimulate its activity. Moreover, DNA2 processes 5′-flaps in the mitochondrial long-patch base excision repair (LP-BER) pathway [69]. Due to its high guanine content, the heavy strand of the mitochondrial genome harbors a higher intrinsic capability to form G-quadruplexes (G4) compared to the nuclear genome [70]. These G4 structures are associated with known deletion breakpoints in the mitochondrial genome [71]. Since knockdown of DNA2 in our screening led to the accumulation of Set2 mtDNA, we speculate that decreased DNA2-mediated cleavage prevents rearrangements at such breakpoints. The other nucleases identified in the screening have not been linked with DSB repair. Rather, EXOG and MGME1 are, in concert with ENDOG, involved in mtDNA degradation [20,72,73] driven by ROS-induced DNA damage [24,74]. This can explain the enrichment of Set2 mtDNA under ROS conditions in MGME1 and EXOG knockdown cells. This effect depends on APE1, which is reminiscent of the observation that the APE1-generated incision is necessary for further nucleolytic processing by ENDOG in the nucleus [28]. Overall, nucleases identified in this screening may promote deleterious events in mtDNA via either rearrangements at replication impediments or degradation of irreparably damaged copies. 

Elevated mtDNA levels after down-regulation of a nuclease that may attack this DNA are quite plausible. Conversely, at first sight, low mtDNA levels seem to be contradictory to reduced expression of a polymerase that may synthesize part or all this DNA. However, knockdown of each single DNA polymerase potentially acting in mitochondria caused a remarkable increase of Set2 mtDNA, namely up to 8.8-fold. In this context, it is of interest that POLG, i.e., the well-established replicative polymerase in mitochondria, has also been connected with degradation of mtDNA to remove abnormal mtDNA species [20,21]. Nissanka and colleagues [21] showed that the exonuclease domain of POLGA is necessary for the degradation of mtDNA fragments. Peeva et al. further demonstrated mtDNA degradation in a concerted action of POLGA 3′-5′ exonuclease and complexed MGME1 5′-3′ exonuclease [20]. Intriguingly, these authors observed that deficiency of the POLGA exonuclease activity leads to persistence of linear fragments comprising the ND1 gene, which is part of the Set2 region [21]. POLGB knockdown was expected to exert the same effect as POLGA knockdown, as POLGB is required to protect POLGA from protein degradation, i.e., to stabilize POLGA through holo-enzyme complex formation [75]. Although the precise mechanisms of DSB formation triggering POLG-dependent mtDNA degradation have not been clarified, ROS-induced DNA lesions and replication stalling have been suggested to play an upstream role [20]. In our screening, we purposefully cultivated the cells in galactose media, enforcing OXPHOS, which leads to an increase of ROS levels in the cell [49,50]. Inhibition of APE1 caused a highly significant 4.6-fold accumulation of Set2 mtDNA, suggesting that incisions of this BER enzyme at abasic sites play a major role in initiating Set2 mtDNA degradation. Simultaneous knockdown of the screening hits from either the nuclease or polymerase groups did not induce an additional rise of Set2 levels, but rather a decrease for some polymerases. We thus propose that the accumulation of BER intermediates promotes deleterious events in mtDNA through the action of the identified nucleases and polymerases. 

POLG is considered the only replicative polymerase in mitochondria [76], but increasing evidence indicates that other polymerases also localize to mitochondria and have a functional role within this organelle [15,36]. POLQ has been shown to play a role in repairing DSBs by MMEJ in the nucleus [77] and in vitro [78]. It is possible that POLQ is also involved in MMEJ to repair ROS-induced DSB [74] in mitochondria, leading to rearrangements and deletions [66,67]. The knockdown of POLQ could lead to a reduction in MMEJ-driven deletions resulting in an upregulation of Set2 mtDNA. Ray and colleagues [79] showed that POLB plays a role in alternative non-homologous end-joining (aNHEJ). It is conceivable that POLB is not only involved in mitochondrial short-patch BER (SP-BER) [14,80] but also in error prone aNHEJ in mitochondria, whereby its depletion results in an accumulation of Set2 mtDNA. 

In this group of top screening hits, knockdown of POLZ caused the most pronounced and highly significant increase in Set2 mtDNA. A MLS at the N-terminus of POLZ was predicted in silico, and its mitochondrial import function verified via analysis of a GFP fusion protein in cellulose [36]. These earlier findings suggesting mitochondrial localization of POLZ were supported by our immunofluorescence confocal microscopy data demonstrating (i) localization of POLZ in TOM22-labeled mitochondria, (ii) reduced extranuclear POLZ signals after inhibition of mitochondrial protein import, and (iii) reduced foci numbers in the proximity ligation assay tracking POLZ in complex with the mitochondrial matrix protein TFAM. Supporting the idea that POLZ not only localizes to but also executes specific functions in mitochondria, we observed upon POLZ knockdown (i) a reduced content of TOM22-labeled mitochondria, (ii) aberrant accumulation of mtDNA in the siRNA screening, and (iii) excess mtDNA synthesis initiation. These pieces of evidence were obtained under conditions of enforced OXPHOS and are therefore reminiscent of previous findings reported upon ENDOG knockdown [29]. In analogy, we conclude that POLZ plays a role in removing oxidatively damaged mtDNA in concert with APE1, which is compensated by the synthesis of new mtDNA copies. In agreement with such a role of POLZ under conditions of oxidative stress, we observed more pronounced mitochondrial POLZ signals in the galactose versus glucose medium. 

Contrary to our findings, i.e., POLZ-dependent degradation of oxidatively damaged mtDNA, Singh et al., 2015 observed protection of the mitochondrial genome upon organelle-specific expression of POLZ. However, unlike our study, which focused on the 6.9 kb Set2 mtDNA, and also addressed the sensitivity to oxidative damage, these authors analyzed a comparatively small (1 kb) region located within Set1. Additionally, these authors applied UV radiation to generate DNA lesions in the mtDNA, while we compared conditions with and without oxidative stress originating from endogenous OXPHOS. Interestingly, when we investigated the formation of a 4kb deletion, called Del3895, within the Set2 region, and previously reported to arise in an UV-inducible manner [43,59,60], we detected POLZ-mediated formation of Del3895. However, in contrast to these previous reports, POLZ was involved in Del3895 formation without UV treatment and only in cells in which mitochondrial ROS was quenched. Moreover, POLZ depletion resulted in an increase of Set2 mtDNA in our screening. We thus propose that POLZ plays an additional role in deleterious rearrangements within the Set2 region of mtDNA in the absence of oxidative damage. 

A clue to the mechanism underlying POLZ-dependent Del3895 formation could come from the fact that POLZ is thought to bind to difficult-to-replicate sites such as G4 structures already arising in the absence of exogenous stress [81]. One of the direct repeat regions (located in the NCR region) at the extremities of the 3895 bp deletion was shown to overlap with a G4 structure, causing stalling of POLG [82]. POLG is known to interact with POLZ [36], so POLG may contribute to the recruitment of POLZ to stalled replication forks to promote TLS [83,84]. As recently shown for nuclear POLZ activities, POLZ-mediated TLS at damaged sites seems to be coupled with PRIMPOL-mediated repriming, transiently exposing single-stranded DNA gaps [85]. In mitochondria, PRIMPOL was demonstrated to exhibit primase activity [86] and reinitiate stalled mtDNA replication [87]. All these DNA structures, i.e., G4, stalled mitochondrial DNA replication, and gaps, are potential targets of nucleases, and may therefore become substrates for rearrangements, such as leading to Del3895. In line with these notions, POLZ has been reported to be involved in microhomology-mediated break-induced replication (MMBIR) [88,89,90], which is a possible mechanism for Del3895 formation. Similarly, POLQ, i.e., another hit from the polymerase group in our screening, with key functions in MMEJ, is a likely candidate for a role in the rearrangements leading to Del3895.

Under conditions of oxidative stress, we did not observe a dependency of Del3895 formation on POLZ. However, the accumulation of the larger Set2 region was antagonized by POLZ and APE1. In our screening, we applied APE1inhIII, which blocks APE1 binding to abasic sites and their cleavage [91]. In our previous study, the same APE1inhIII prevented downstream cleavage of a difficult-to-replicate site in the nuclear genome by ENDOG under conditions of replicative stress [28]. Given that not only APE1 binds to G4 sequences when oxidatively damaged [92,93], but also POLZ is thought to act on G4 [81], we propose that POLZ promotes APE1 binding to damaged and/or difficult-to-replicate sequences in the Set2 region, accelerating their cleavage. APE1 is known to interact with POLB, and in this complex might act as a cryptic exonuclease, since POLB, similar to POLZ [83], lacks this function [94,95]. Of note, APE1 was previously found to compete with TFAM for binding to AP lesions and was suggested to regulate the balance between mtDNA repair and degradation [25]. In support of such an indirect route of regulating the turnover of mtDNA, we indeed noticed an association of POLZ and TFAM in mitochondria.

Altogether, our study provides evidence for the role of POLZ in mtDNA maintenance regulation in a pathway depending on APE1 activity. However, we are aware of the complexity of the observed phenomenon that may not only involve mtDNA repair and turnover, but also adaptive replication mode changes [96]. Further investigations into the mechanism by which POLZ regulates mtDNA maintenance are necessary. POLZ harbors several interaction partners, such as POLG, and has biochemical activities, such as recognition of secondary DNA structures, TLS, and MMBIR. These properties can provide guidance for the design of future studies to unravel the molecular details of the newly identified role of POLZ in mtDNA maintenance. These studies will be of utmost importance for fully understanding nuclear and mitochondrial POLZ functions, which are of particular relevance for the development of POLZ-inhibitory compounds for the treatment of homologous recombination-deficient tumors [85].

## Data Availability

Not applicable.

## Supplementary Materials

The following supporting information can be downloaded at: www.mdpi.com/article/10.3390/genes13050879/s1, Table S1: Candidates of the siRNA screening library; Figure S1: Impact of APE1inhIII on mtDNA maintainance; Figure S2: Knockdown efficiency; Figure S3: Del3895 formation after ENDOG or POLGA knockdown; Figure S4: Effect of mtDNA replication inhibition; Figure S5: POLZ localizes to mitochondria; Figure S6: POLZ level depends on ROS; Figure S7: POLZ depeletion reduces mitochondrial mass.
