# Supplementary material for: Polymerase ζ Is Involved in Mitochondrial DNA Maintenance Processes in Concert with APE1 Activity
Source: Genes (Basel). 2022 May 13;13(5):879. doi: 10.3390/genes13050879 (PMC9141751; doi:10.3390/genes13050879)
Supplement: Supplementary file 1 [file genes-13-00879-s001.zip › genes-1653523-supplementary.pdf]

genes

MDPI

Supplement:

Table S1. Candidates of the siRNA screening library.

Gene Name	GeneID	Protein Name	Group
AAG/MPG	4350	DNA-3-methyladenine glycosylase	Base excision repair (BER)
APEX2	27301	DNA-(apurinic or apyrimidinic site) endonuclease 2	Nucleases
APTX	54840	Aprataxin	Single-strand break repair (SSB-repair)
BRCA1	672	Breast cancer type 1 susceptibility protein	Double-strand break repair (DSB-repair)
CHIP/STUB1	10237	E3 ubiquitin-protein ligase CHIP	ENDOG interacting
CSA/ERCC8	1161	DNA excision repair protein ERCC-8	Single-strand break repair (SSB-repair)
CSB/ERCC6	2074	DNA excision repair protein ERCC-6	Single-strand break repair (SSB-repair)
CTIP/RBBP8	5932	DNA endonuclease RBBP8	Nucleases
DNA2	1763	DNA replication ATP-dependent helicase/nuclease DNA2	Nucleases/Base excision repair (BER)
ELAC2	60528	Zinc phosphodiesterase ELAC protein 2	RNA Processing
ENDOG	2021	Endonuclease G, mitochondrial	Nucleases
EXOG	9941	Nuclease EXOG, mitochondrial	Nucleases/Base excision repair (BER)
FEN1	2237	Flap endonuclease 1	Nucleases/Base excision repair (BER)
GRSF1	2926	G-rich sequence factor 1	RNA Processing
HSPA9	3313	Stress-70 protein, mitochondrial	ENDOG interacting
LIG3	3980	DNA ligase 3	Base excision repair (BER)/mtDNA Replication
MGME1	92667	Mitochondrial genome maintenance exonuclease 1	Nucleases/mtDNA Replication
MRE11	4361	Double-strand break repair protein MRE11	Double-strand break repair (DSB-repair)
MRPP3/PRORP	9692	Mitochondrial ribonuclease P catalytic subunit	RNA Processing
MTERF1	7978	Transcription termination factor 1, mitochondrial	Mitochondrial Transcription
MUTYH	4595	Adenine DNA glycosylase	Base excision repair (BER)
NEIL1	79661	Endonuclease 8-like 1	Base excision repair (BER)
NEIL2	252969	Endonuclease 8-like 2	Base excision repair (BER)
NTHL1	4913	Endonuclease III-like protein 1	Base excision repair (BER)
OGG1	4968	N-glycosylase/DNA lyase	Base excision repair (BER)
PARP1	142	Poly [ADP-ribose] polymerase 1	Single-strand break repair (SSB-repair)
PNKP	11284	Bifunctional polynucleotide phosphatase/kinase	Base excision repair (BER)
PNPASE/PNPT1	87178	Polyribonucleotide nucleotidyltransferase 1, mitochondrial	RNA Processing
POLB	5423	DNA polymerase beta	Polymerases/Base excision repair (BER)
POLGB/POLG2	11232	DNA polymerase subunit gamma-2, mitochondrial	Polymerases/mtDNA Replication/ Base excision repair (BER)
POLGA/POLG1	5428	DNA polymerase subunit gamma-1	Polymerases/mtDNA Replication/Base excision repair (BER)
POLQ	10721	DNA polymerase theta	Polymerases
POLRMT	5442	DNA-directed RNA polymerase, mitochondrial	Polymerases/mtDNA Replication & Transcription
POLZ/REV3L	5980	DNA polymerase zeta catalytic subunit	Polymerases
PRIMPOL	201973	DNA-directed primase/polymerase protein	Polymerases
PTCD2	79810	Pentatricopeptide repeat-containing protein 2, mitochondrial	RNA Processing
RAD51	5888	DNA repair protein RAD51 homolog 1	Double-strand break repair (DSB-repair)
RAD51C	5889	DNA repair protein RAD51 homolog 3	Double-strand break repair (DSB-repair)
REXO2	25996	Oligoribonuclease, mitochondrial	RNA Processing
RMRP	6023	RNA component of mitochondrial RNA processing endoribonuclease	RNA Processing
RNASEH1	246243	Ribonuclease H1	Mitochondrial Replication/RNA Processing

SSBP1	6742	Single-stranded DNA-binding protein, mitochondria (mt-SSB)	Mitochondrial Replication
SUV3/SUPV3L1	6832	ATP-dependent RNA helicase SUPV3L1, mitochondrial	RNA Processing
TDP1	55775	Tyrosyl-DNA phosphodiesterase 1	Single-strand break repair (SSB-repair)
TEFM	79736	Transcription elongation factor, mitochondrial	Mitochondrial Transcription
TET2	54790	Methylcytosine dioxygenase TET2	ENDOG interacting
TFAM	7019	Transcription factor A, mitochondrial	Mitochondrial Transcription
TFB2M	64216	Dimethyladenosine transferase 2, mitochondrial	Mitochondrial Transcription
TOP3A	7156	DNA topoisomerase 3-alpha	Mitochondrial Replication
TP53	7157	Cellular tumor antigen p53	Single-strand break repair (SSB-repair)
TRAP1	10131	Heat shock protein 75 kDa, mitochondrial	ENDOG interacting
TWNK	56652	Twinkle protein, mitochondrial	Mitochondrial Replication
UNG	7374	Uracil-DNA glycosylase	Base excision repair (BER)
XRCC3	7517	DNA repair protein XRCC3	Double-strand break repair (DSB-repair)
XRCC5/ KU80	7520	X-ray repair cross-complementing protein 5	Double-strand break repair (DSB-repair)
XRCC6/ KU70	2547	X-ray repair cross-complementing protein 6	Double-strand break repair (DSB-repair)
YB1/ YBX1	4904	Y-box-binding protein 1	mismatch repair (MMR)

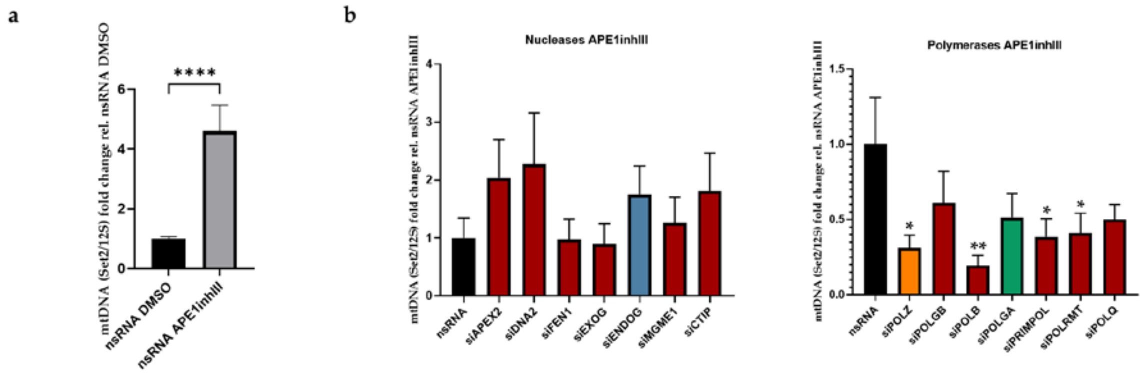

Figure S1. Impact of APE1inhIII on mtDNA maintenance: nsRNA transfected control cells were treated with DMSO or 15 µM APE1inhIII for 5 h after 48 h cultivation in galactose medium (a) PCR products of the 7 kb long mtDNA Set2 region amplified from total DNA were quantified, normalized to the sample-specific values for the 0.2 kb short mitochondrial 12S region and plotted relative (rel.) to the mean values for nsRNA transfected samples per experiment (n = 56 from 28 independent experiments). (**b**) HeLa cells were grown in galactose medium and subjected to siRNA-mediated knockdown of indicated candidate proteins associated with mitochondrial functions for 48 h and treated with 15 µM APE1inhIII for 5 h. Set2/12S mtDNA was plotted relative to nsRNA (n = 8 from 4 independent experiments). All values are means ± SEM (* $p \leq 0.05$, ** $p \leq 0.01$ and **** $p \leq 0.0001$).

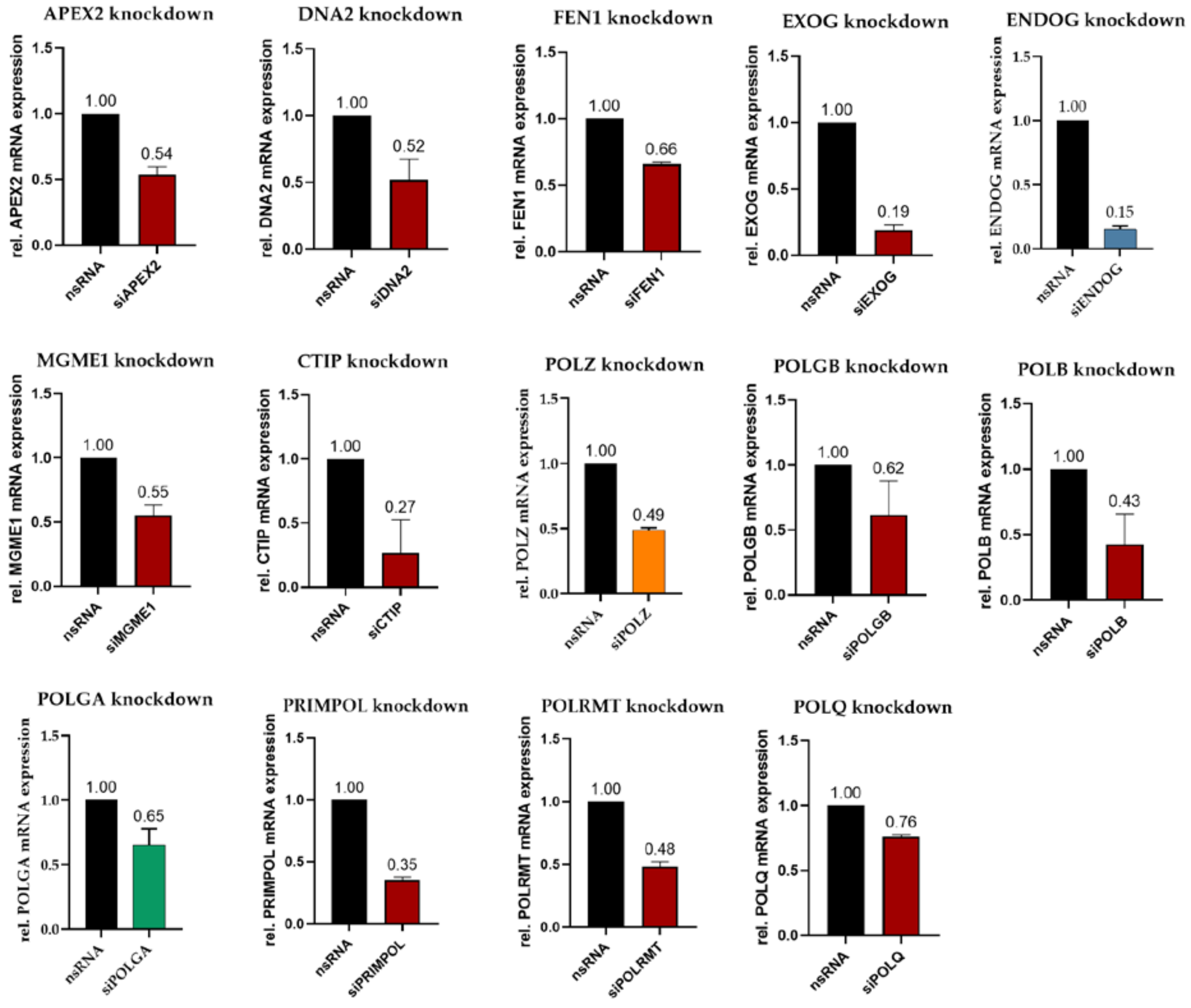

Figure S2. Knockdown efficiency: qPCR-based quantification of the gene expression of the group of nucleases and polymerases in Figure 3b after transfection with 1nM nsRNA or 1nM specific siRNA. All values are means ±SD of n = 4 experiments from 2 independent mRNA isolations.

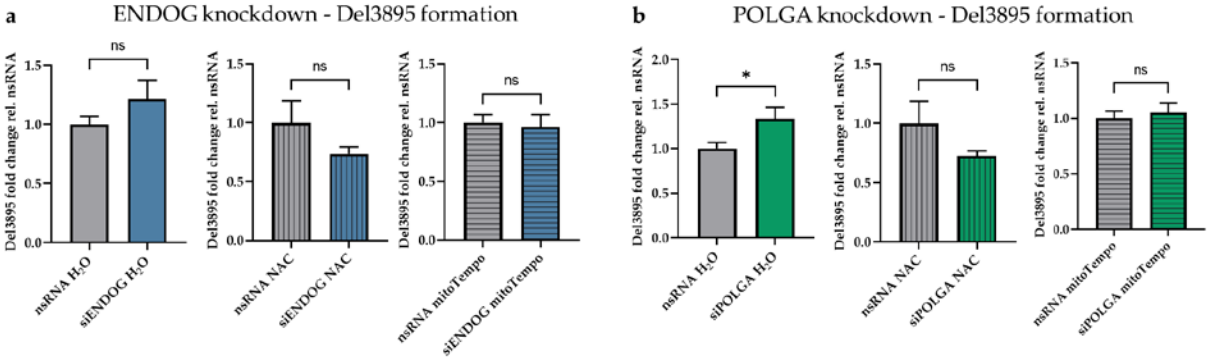

Figure S3. Del3895 formation after ENDOG or POLGA knockdown**:** Quantification of Del3895 deletion product from HeLa cells cultured in galactose medium, siRNA-mediated knockdown for 48 h of ENDOG (**a**) or POLGA (**b**) plus 24 h treatment with 100 µM mitoTempo or 2 mM NAC as outlined in Figure 4a (n = 10 from 5 independent experiments). All values are means ± SEM (* $p \leq 0.05$).

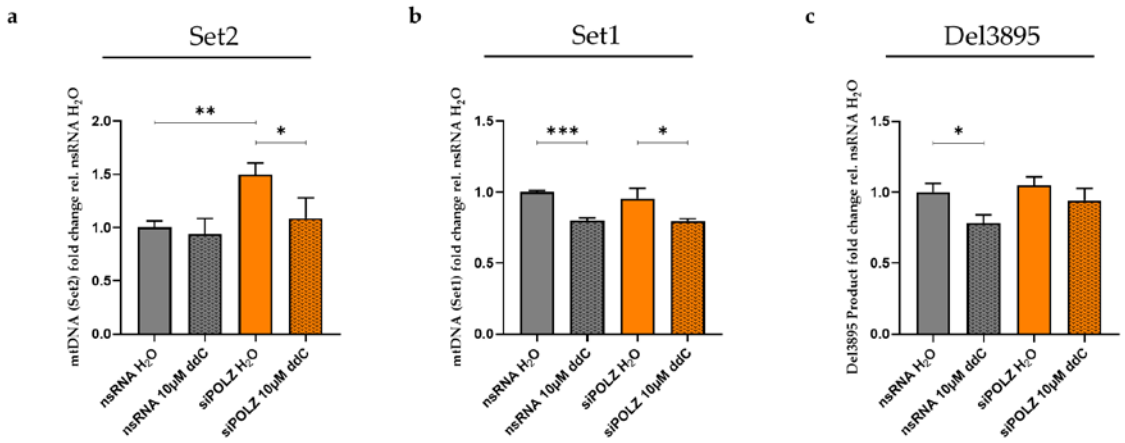

Figure S4. Effect of mtDNA replication inhibition. Hela cells were cultured in galactose medium and POLZ knockdown was performed for 48 h. Total DNA was isolated after treatment with H_2O or 10 µM ddC for 24 h. Quantified Set2 PCR products (**a**), Set1 PCR products (**b**) and Del3895 product formation (**c**) were plotted relative to nsRNA H_2O control values for cells transfected with nsRNA (grey bars) or siPOLZ (orange bars). All values are means ± SEM (n = 7–8 of 4 independent experiments) (* $p \le 0.05$, ** $p \le 0.01$ and *** $p \le 0.001$).

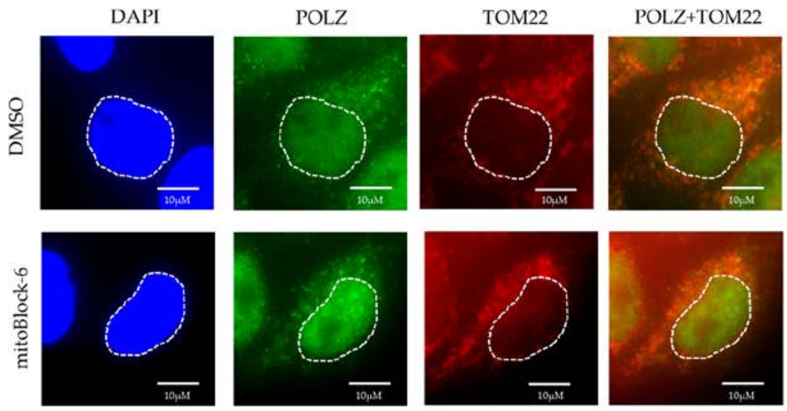

Figure S5. POLZ localizes to mitochondria**.** Representative images of POLZ fluorescence intensity in HeLa cells treated for 48 h with DMSO or 10 µM mitoBlock-6 showing colocalization with mitochondrial marker TOM22. scale bar = 10µM.

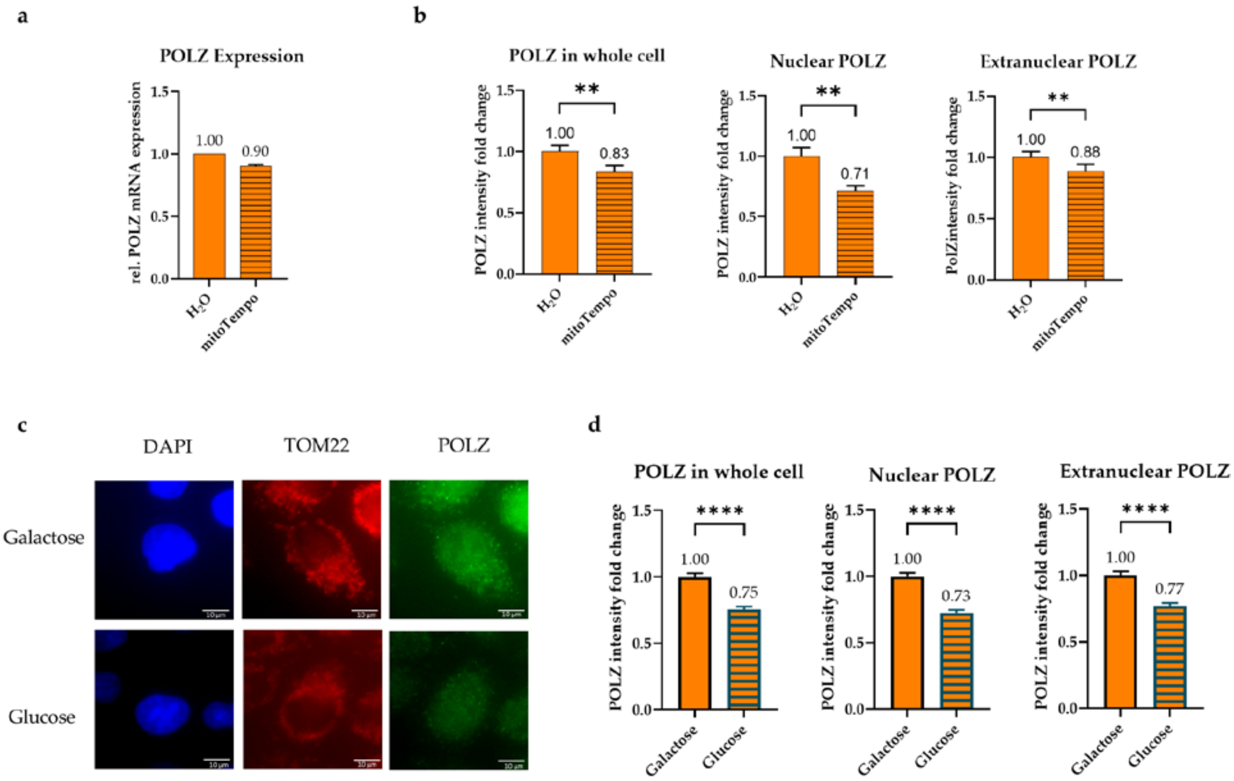

Figure S6. POLZ level depends on ROS: Quantification of POLZ expression by qRT-PCR in HeLa cells cultured in galactose medium and treated with H_2O or 100 µM mitoTempo for 24 h (**a**). Fluorescence-based quantification of POLZ signal intensities in the whole cell, nucleus or extranuclear regions, n = 74–79 from 2 independent experiments (**b**). Representative microscopic images of HeLa cells cultured 72 h in either galactose or glucose media stained for mitochondrial TOM22 and POLZ (**c**) and the quantification of POLZ signal in the whole cell, nucleus and extranuclear regions, n = 169-174 cells from 3 independent experiments (**d**). All values are means ± SEM, (** $p \leq 0.01$, **** $p \leq 0.0001$), scale bar = 10µM.

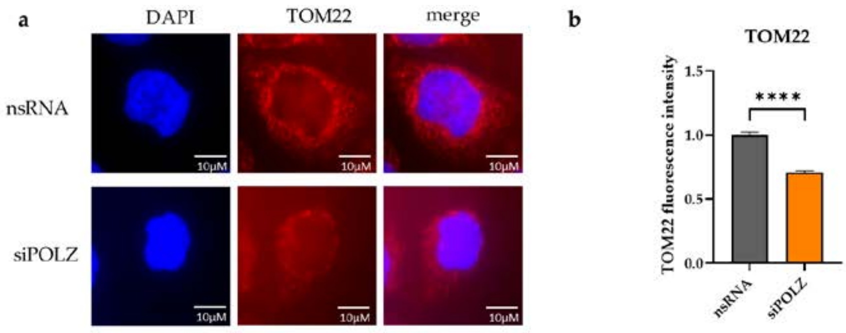

Figure S7. POLZ depletion reduces mitochondrial mass: Representative immunofluorescence images of TOM22 intensity in HeLa cells transfected with nsRNA or siPOLZ for 48 h (**a**) scale bar = 10 µM and their quantification (**b**). All values are ± SEM from two independent experiments n = 189–204 cells (**** $p \leq 0.0001$).
